# Evaluation of the impact of surgical aortic valve replacement on short-term cardiovascular and cerebrovascular controls through spontaneous variability analysis

**DOI:** 10.1371/journal.pone.0243869

**Published:** 2020-12-10

**Authors:** Alberto Porta, Angela Fantinato, Vlasta Bari, Francesca Gelpi, Beatrice Cairo, Beatrice De Maria, Enrico Giuseppe Bertoldo, Valentina Fiolo, Edward Callus, Carlo De Vincentiis, Marianna Volpe, Raffaella Molfetta, Marco Ranucci

**Affiliations:** 1 Department of Biomedical Sciences for Health, University of Milan, Milan, Italy; 2 Department of Cardiothoracic, Vascular Anesthesia and Intensive Care, IRCCS Policlinico San Donato, Milan, Italy; 3 IRCCS Istituti Clinici Scientifici Maugeri, Milan, Italy; 4 Clinical Psychology Service, IRCCS Policlinico San Donato, Milan, Italy; 5 Department of Cardiac Surgery, IRCCS Policlinico San Donato, Milan, Italy; 6 Department of Cardiac Rehabilitation, IRCCS Policlinico San Donato, Milan, Italy; Scuola Superiore Sant'Anna, ITALY

## Abstract

We assessed the effect of surgical aortic valve replacement (SAVR) on cardiovascular and cerebrovascular controls via spontaneous variability analyses of heart period, approximated as the temporal distance between two consecutive R-wave peaks on the electrocardiogram (RR), systolic, diastolic and mean arterial pressure (SAP, DAP and MAP) and mean cerebral blood flow (MCBF). Powers in specific frequency bands, complexity, presence of nonlinear dynamics and markers of cardiac baroreflex and cerebral autoregulation were calculated. Variability series were acquired before (PRE) and after (POST) SAVR in 11 patients (age: 76±5 yrs, 7 males) at supine resting and during active standing. Parametric spectral analysis was performed based on the autoregressive model. Complexity was assessed via a local nonlinear prediction approach exploiting the k-nearest-neighbor strategy. The presence of nonlinear dynamics was checked by comparing the complexity marker computed over the original series with the distribution of the same index assessed over a set of surrogates preserving distribution and power spectral density of the original series. Cardiac baroreflex and cerebral autoregulation were estimated by assessing the transfer function from SAP to RR and from MAP to MCBF and squared coherence function via the bivariate autoregressive approach. We found that: i) orthostatic challenge had no effect on cardiovascular and cerebrovascular control markers in PRE; ii) RR variance was significantly reduced in POST; iii) complexity of SAP, DAP and MAP variabilities increased in POST with a greater likelihood of observing nonlinear dynamics over SAP compared to PRE at supine resting; iv) the amplitude of MCBF variations and MCBF complexity in POST remained similar to PRE; v) cardiac baroreflex sensitivity decreased in POST, while cerebrovascular autoregulation was preserved. SAVR induces important changes of cardiac and vascular autonomic controls and baroreflex regulation in patients exhibiting poor reactivity of cardiovascular regulatory mechanisms, while cerebrovascular autoregulation seems to be less affected.

## Introduction

Since sympathetic activation and vagal withdrawal lead to the decrease of the variability of heart period, approximated as the temporal distance between two consecutive R-wave peaks on the electrocardiogram (RR), and to the increase of the fluctuations of systolic arterial pressure (SAP), the RR and SAP variances have been exploited to infer vagal and sympathetic neural controls [1–6]. Spectral analysis allowed a more precise link of RR and SAP variabilities with the state of the autonomic nervous system given that oscillations in the high frequency (HF, from 0.15 to 0.4 Hz) band of the RR series are more specifically associated to vagal control directed to the sinus node [1–6], while those in the low frequency (LF, from 0.04 to 0.15 Hz) band of the SAP series are more specifically linked to sympathetic control directed to the vessels [4–6]. Complexity and nonlinear content of the RR and SAP variability series are frequently explored to gain insight into peculiar features of autonomic regulatory mechanisms that cannot be fully described by power spectral density [7–13]. Complexity of the cardiac control is reduced during sympathetic activation and vagal withdrawal [7, 8]. The development of nonlinear dynamics appears to be favored by an enhancement of the vagal control [9–12] and by a weakened action of the cardiac baroreflex [13]. Also the different response of regulatory mechanisms to positive or negative SAP variations and circuits imposing a certain degree of cardiorespiratory phase coupling might play an important role in producing nonlinear RR variability patterns [14–16].

The integrative regulation of cerebral blood flow (CBF) comprising chemoreflex, neuronal metabolism, cerebral autoregulation and autonomic control [17] is routinely assessed via non-interventional techniques based on the analysis of the spontaneous variability of physiological variables related to cerebral circulatory system such as mean arterial pressure (MAP), acquired via volume-clamp photoplethysmography from the middle finger [4], and mean CBF (MCBF) [18], estimated via transcranial Doppler device from middle cerebral arteries [19, 20]. The assessment of the MAP and MCBF powers and of the MCBF-MAP dynamical relation in specific frequency bands is the basis for the evaluation of the ability of cerebral vasculature to buffer MCBF via suitable modifications of cerebral resistance [18]. Given the important contribution of the autonomic nervous system to integrative regulation of CBF [17, 21], the concurrent evaluation of cerebrovascular control with autonomic markers derived from RR and SAP variability series might provide more insight into cerebrovascular control mechanisms and complex interactions among cardiovascular and cerebrovascular regulations.

A deep analysis of the impact of cardiac surgery on cardiovascular and cerebrovascular controls is missing. Information is mainly limited to cardiovascular control. Indeed, spectral analysis of RR variability suggested that cardiac surgery depresses vagal control [22–25] as well as baroreflex function [26], thus exposing patients to a higher risk of postoperative adverse events such as atrial fibrillation and acute kidney dysfunction [26, 27]. Complexity analysis of RR and SAP variability series was less frequently performed after cardiac surgery. Complexity analysis of RR and SAP variability series suggested a more variable response to cardiac surgery across individuals and groups [26, 28]. Information about the impact of cardiac surgery on cerebrovascular control is even more limited [29]. Given the postoperative impairment of the autonomic control [22–26] and the relevant impact of the autonomic function on integrative regulation of CBF [17, 21], we hypothesize a dramatic influence of cardiac surgery on the magnitude of MAP and MCBF variations and/or MCBF-MAP dynamical relation. The assessment of cerebrovascular control after cardiac surgery might provide more information about the patient’s post-operative state and, if this characterization was carried out in association with that of the cardiovascular one, the description might be more complete and insightful.

The aim of this study is to characterize cardiovascular and cerebrovascular controls via spectral and complexity analyses and to test the presence of nonlinear patterns from variability series recorded before and after surgical aortic valve replacement (SAVR). Variability of RR, SAP, and diastolic arterial pressure (DAP) were analyzed to infer the state of cardiac and vascular controls, while the variability of MCBF and MAP was assessed to infer that of cerebrovascular regulation. Nonlinear dynamics were detected through a local nonlinear prediction marker [10, 30] in association with a surrogate data approach [10, 31]. The analysis was completed with the description of the baroreflex control and cerebral autoregulation via the transfer function method applied to SAP and RR variability series [32] and MAP and MCBF variability series [18] respectively. Preliminary results were presented at the 11^th^ meeting of the European Study Group of the Cardiovascular Oscillations [33] and at the 42^nd^ Annual International Conference of the Engineering in Medicine and Biology Society [34].

## Methods

### Ethics statement

The study was in keeping with the Declaration of Helsinki. The study was approved by the ethical review board of the San Raffaele Hospital, Milan, Italy (approval number: 68/int/2018; approval date: 05/04/2018) and authorized by the Policlinico San Donato, San Donato Milanese, Milan, Italy (authorization date: 13/04/2018). Written signed informed consent was obtained from all subjects.

### Population and experimental protocol

We enrolled 11 patients (age: 76±5 yrs, 7 males) undergoing SAVR at the IRCCS Policlinico San Donato, San Donato Milanese, Milan, Italy. Demographic and clinical data of the SAVR group were reported in Table 1. They did not feature either atrial fibrillation, overt autonomic nervous system pathologies or cerebrovascular diseases. We acquired electrocardiogram (ECG) from lead II (BioAmp FE132, ADInstruments, Australia), non-invasive finger arterial pressure (AP) by volume-clamp photoplethysmography (CNAP Monitor 500, CNSystems, Austria), respiration (RESP) via a thoracic piezoelectric belt (ADinstruments, Australia) and CBF velocity via a transcranial Doppler device (Multi-Dop X, DWL, San Juan Capistrano, CA, USA) from the left or right middle cerebral artery. Signals were sampled at 400 Hz through a commercial acquisition system (Power Lab, ADInstruments, Australia). Signals were recorded 1 day before SAVR (PRE) and within 7 days after SAVR (POST). Acquisition sessions comprised recordings at rest in supine position (REST) and during active standing (STAND). REST and STAND lasted 10 minutes and REST always preceded STAND. In PRE REST and STAND sessions were carried out in all subjects. In POST REST was performed in 8 individuals and STAND in 6 subjects due to the physical and psychological debilitation of some patients. ECG and AP were recorded in all the subjects present in a given experimental session. Conversely, CBF was recorded in PRE in 10 subjects out of 11 at REST and in 8 individuals out of 11 during STAND and in POST in 6 subjects out of 8 at REST and in 4 individuals out of 6 during STAND. These figures were due to the difficulty in locating either left or right middle cerebral artery.

**Table 1:** Clinical and demographic markers of SAVR subjects.

marker	SAVR (n = 11)
Age [yrs]	76 ± 5
Gender [male]	7 (64)
Weight [kg]	72.5 ± 11.5
BMI [kg·m^-2^]	26.3 ± 3.9
Congestive heart failure	0 (0)
Recent myocardial infarction	0 (0)
Previous cerebrovascular events	0 (0)
LVEF [%]	59.9 ± 7.0
Diabetes	1 (9)
COPD	1 (9)
Serum creatinine [mg·dl^-1^]	0.92 ± 0.19
Hypertension	9 (82)
HCT [%]	40.6 ± 4.9
ACE inhibitors	7 (64)
Beta-blockers	6 (55)
Diuretics	4 (36)
Calcium antagonists	2 (18)
Antiarrhythmic drugs	0 (0)
Combined intervention	8 (73)
EuroSCORE II	3.0 ± 2.6
CPB time [minutes]	109.6 ± 43.3
Nadir temperature on CPB [°C]	33.6 ± 1.5
Catecholamine administration	3 (27)
Mechanical ventilation time [hours]	11.2 ± 6.2
ICU stay [days]	2.1 ± 1.0
Hospital stay [days]	8.3 ± 3.8
Postoperative atrial fibrillation	6 (55)
Postoperative arrhythmias	2 (18)
Postoperative low cardiac output syndrome	2 (18)
Postoperative stroke	0 (0)
Postoperative acute kidney injury	0 (0)

SAVR = surgical aortic valve replacement; BMI = body mass index; LVEF = left ventricular ejection fraction; COPD = chronic obstructive pulmonary disease; HCT = hematocrit; ACE = angiotensin converting enzyme; EuroSCORE = European System for Cardiac Operative Risk Evaluation; CPB = cardiopulmonary bypass; ICU = intensive care unit. Continuous data are presented as mean ± standard deviation and categorical data as number (percentage).

### Extraction of beat-to-beat variability series and time domain indexes

After detecting the R-wave peaks from the ECG with a classical method based on a threshold applied to the first derivative, the *i*th RR, where *i* is the progressive cardiac beat number, was derived as the time distance between the (*i*-1)th and the *i*th cardiac beat. The *i*th SAP and DAP were measured, respectively, as the AP maximum within the *i*th RR and the AP minimum following the *i*th SAP. The *i*th MAP was obtained as the integral of AP between the (*i*-1)th and *i*th DAP fiducial points and by dividing the result by the interdiastolic time interval. The *i*th RESP was obtained by sampling RESP signal at the apex of the first R-wave peak delimiting the *i*th RR. The *i*th MCBF was obtained as the integral of CBF between the (*i*-1)th and *i*th minima detected over CBF and closest in time to (*i*-1)th and *i*th DAP fiducial points and by dividing the result by the time distance between the two minima [35]. The RR, SAP, DAP, MAP and MCBF series were manually checked and corrected in case of missing beats or misdetections. Effects of ectopic beats or isolated arrhythmic events were mitigated via linear interpolation. Spectral, cross-spectral, complexity analyses as well as detection of nonlinear dynamics were carried out directly over synchronous sequences lasting 256 consecutive beats randomly selected within the whole recordings. In time domain we computed the means, indicated as μ_RR_, μ_SAP_, μ_DAP_, μ_MAP_ and μ_MCBF_, and the variances, denoted with σ^2^_RR_, σ^2^_SAP_, σ^2^_DAP_, σ^2^_MAP_ and σ^2^_MCBF_. Means and variances were expressed, respectively, in ms, mmHg, mmHg, mmHg and cm·s^-1^ and ms^2^, mmHg^2^, mmHg^2^, mmHg^2^ and cm^2^·s^-2^. After computing the mean, the series were linearly detrended before computing variance.

### Univariate model-based frequency domain analysis

Univariate model-based frequency domain analysis was carried out via a traditional parametric power spectral method (see *S1 Appendix*). Briefly, variability series were described as a realization of an autoregressive (AR) process modeling the variation of the most recent value of the series about its mean as a linear combination of *p* past changes weighted by constant coefficients plus a sample drawn from a realization of a zero mean white noise, where *p* is the order of AR model [36, 37]. The coefficients of the AR model and the variance of the white noise were identified directly from the series by solving the least squares problem via Levinson-Durbin recursion [36]. The number of coefficients *p* was chosen according to the Akaike’s figure of merit in the range from 8 to 16 [38]. Power spectral density was computed from the AR coefficients and from the variance of the white noise according to the maximum entropy spectral estimation approach [36]. The power spectral density was factorized into a sum of terms, referred to as spectral components, the sum of which provides the entire power spectral density [37]. Power spectral decomposition provided the central frequency of the components expressed in normalized frequency units, namely cycles per beat. Central frequency ranged from 0 to 0.5 cycles per beat and was converted into Hz by dividing the value by the average sampling period *T* of the variability series, i.e. *T* = μ_RR_, expressed in s [37]. A spectral component was attributed to a specific frequency band if its central frequency lay in that band. If multiple spectral components were found to belong to the same frequency band, their powers were summed up and the weighted central frequency was computed.

### Bivariate model-based frequency domain analysis

Bivariate model-based frequency domain analysis was carried out via a traditional parametric cross-spectral method (see *S1 Appendix*). This method allows the assessment of the input-output relation in the frequency domain (i.e. the transfer function) between two series when one is assumed to be the cause and the other is taken as the effect [32]. The dynamics of input and output series about their mean values were jointly described as a bivariate AR process [39] modeling the variation of the most recent value of one series about its mean as a linear combination of *p* past changes of the same series and *p* past variations of the other series weighted by constant coefficients plus a sample drawn from a realization of a zero mean white noise, where *p* is the order of the bivariate AR model. Although the bivariate AR model described the closed loop relation via the representation of the feedforward and feedback arms [37], we focused our attention on a specific direction of interactions by assigning *a priori* one series as the input and the other as the output [32]. The cross-spectrum from the input to the output and the autospectra of both input and output were computed from the coefficients of the bivariate AR model and from the variance of the white noises [39]. The model order *p* was fixed at 10 and the coefficients of the bivariate AR model were identified via least squares approach solved using Cholesky decomposition method [37, 39]. The transfer function (TF) was estimated as the ratio of the cross-spectrum computed from the input to the output to the power spectrum of input series [40]. The TF modulus (TFM) and phase were calculated as a function of the frequency. The TFM could not be negative and was expressed in units being the ratio of the unit of the output to that of the input. The phase was expressed in radians (rad) and ranged between -π and +π, with negative value indicating that the output lagged behind the input. The ratio of the squared cross-spectrum modulus to the product of the power spectra of the input and output series provided the estimation of the squared coherence function (K^2^) as a function of the frequency [40]. The K^2^ was dimensionless and ranged between 0 and 1, where 0 indicated full uncoupling and 1 perfect association between the input and the output. The TFM, phase and K^2^ functions were sampled in correspondence of the frequency where the K^2^ peaked the maximum value within the considered frequency band [41].

### Cardiovascular variability and cardiac baroreflex markers

Cardiovascular regulation was assessed in the low frequency (LF, from 0.04 to 0.15 Hz) and high frequency (HF, from 0.15 to 0.4 Hz) bands [2]. The power of the RR series in the HF band expressed in absolute units (HFa_RR_) was taken as a marker of the vagal modulation directed to the heart [1, 4, 6] and the power of the SAP and DAP series in the LF band expressed in absolute units (LFa_SAP_ and LFa_DAP_) was taken as an index of sympathetic modulation directed to the vessels [5, 6]. The central frequency of the dominant component of the RESP series in the HF band was taken as an estimate of the respiratory rate (f_RESP_). HFa_RR_, LFa_SAP_, LFa_DAP_ and f_RESP_ were expressed in ms^2^, mmHg^2^, mmHg^2^ and Hz respectively. The baroreflex function was assessed through the computation of the TFM from SAP to RR in the LF and HF bands [TFM_RR-SAP_(LF) and TFM_RR-SAP_(HF)], the TF phase from SAP to RR in the LF and HF bands [Ph_RR-SAP_(LF) and Ph_RR-SAP_(HF)] and K^2^ between SAP and RR in the LF and HF bands [K^2^_RR-SAP_(LF) and K^2^_RR-SAP_(HF)]. TFM_RR-SAP_(LF) and TFM_RR-SAP_(HF) were taken as an estimate of the baroreflex sensitivity [32, 40, 42] and expressed in ms·mmHg^-1^. While Ph_RR-SAP_(LF) and Ph_RR-SAP_(HF) were considered markers of the delay/advance of SAP on RR [43], K^2^_RR-SAP_(LF) and K^2^_RR-SAP_(HF) were measures of the RR-SAP coupling strength [44].

### Cerebrovascular variability and cerebral autoregulation indexes

Cerebrovascular regulation was assessed in the very low frequency (VLF, from 0.02 to 0.07 Hz), LF (from 0.07 to 0.2 Hz) and high frequency (HF, from 0.2 to 0.4 Hz) bands [18]. The power of the MAP and MCBF series were expressed in absolute units, namely mmHg^2^ and cm^2^·s^-2^, and labelled VLFa_MAP_, VLFa_MCBF_, LFa_MAP_, LFa_MCBF_, HFa_MAP_ and HFa_MCBF_. The power was expressed in percent units assessed as the ratio of the power expressed in absolute units (multiplied by 100) to the variance. Percent power indexes were labelled VLF%_MAP_, VLF%_MCBF_, LF%_MAP_, LF%_MCBF_, HF%_MAP_ and HF%_MCBF_. The cerebral autoregulation was assessed through the computation of the TFM from MAP to MCBF in the VLF, LF and HF bands [TFM_MCBF-MAP_(VLF), TFM_MCBF-MAP_(LF) and TFM_MCBF-MAP_(HF)], the TF phase from MAP to MCBF in the VLF, LF and HF bands [Ph_MCBF-MAP_(VLF), Ph_MCBF-MAP_(LF) and Ph_MCBF-MAP_(HF)] and K^2^ between MAP and MCBF in the VLF, LF and HF bands [K^2^_MCBF-MAP_(VLF), K^2^_MCBF-MAP_(LF) and K^2^_MCBF-MAP_(HF)]. TFM_MCBF-MAP_(VLF), TFM_MCBF-MAP_(LF) and TFM_MCBF-MAP_(HF) were taken as an estimate of the cerebrovascular autoregulation sensitivity [45] and expressed in cm·s^-1^·mmHg^-1^. While Ph_MCBF-MAP_(VLF), Ph_MCBF-MAP_(LF) and Ph_MCBF-MAP_(HF) were considered markers of the delay/advance of MAP on MCBF [46] and K^2^_MCBF-MAP_(VLF), K^2^_MCBF-MAP_(LF) and K^2^_MCBF-MAP_(HF) were indexes of the MCBF-MAP coupling strength [47].

### Complexity analysis based on local nonlinear prediction

From the series *y* = {*y*_*i*_, *i* = 1,…,*N*}, where *N* is the series length, we built the pattern set ***y*** = {***y***_*i*_ = (*y*_*i*_, *y*_*i-1*_, ···, *y*_*i*-*L*+1_), *i* = *L*,…,*N*}, where *L* is the pattern length and ***y***_*i*_ is an ordered vector of *L* consecutive samples taken from *y*. Given the reference pattern ***y***_*i*_, its *k* nearest neighbors {***y***_*k*_} were searched for and the images of the reference pattern and its *k* nearest neighbors, namely *y*_*i*+1_ and {*y*_*k*+1_}, were retained. We defined the best prediction of image *y*_*i*+1_ of the reference pattern as the weighted average of the images {*y*_*k*+1_} of its *k* nearest neighbors, where the weights were the distances of each *k* nearest neighbor from the reference pattern [30]. The ability to predict was quantified as the complement to 1 of the squared correlation coefficient between the original series and the predicted one [10]. According to [10, 48] *k* was set to 30, the distance was computed using the Euclidian norm and solely the reference vector was excluded from the set of its nearest neighbors. This cost function exhibited a minimum resulting from two contrasting tendencies, namely the ability to predict future values increasing with *L* and the growing dispersion of the patterns in the *L*-dimensional space resulting from the wider and wider volume occupied by the points with *L* [10]. The minimum of this cost function over *L* was taken as normalized complexity index (NCI) [10]. The greater the NCI and the closer to 1, the more unpredictable and complex the series. The smaller the NCI and the closer to 0, the more regular and predictable the series. NCI was computed over all the series and the indexes were labelled NCI_RR_, NCI_SAP_, NCI_DAP_, NCI_RESP_, NCI_MAP_ and NCI_MCBF_.

### Testing the null hypothesis of linear dynamic

We tested the null hypothesis of linear dynamic using a surrogate data approach in connection with a nonlinear index such as NCI [10, 48]. One hundred surrogate series were generated from the original series according to the amplitude-adjusted iteratively-refined Fourier transform method [31]. This method iterates the following steps [31]: i) the Fourier transform is applied to the series; ii) the Fourier phases are substituted with numbers drawn from a uniform distribution bounded between 0 and 2π; iii) the inverse Fourier transform is applied to return in the time domain, thus generating the surrogate; iv) the distribution of the surrogate is constrained to be exactly equal to that of the original series by substituting any sample of the surrogate with that of the original series that occupies the same position according to a rank ordering procedure applied to both surrogate and original series; v) all the previous steps are repeated until the power spectrum of the surrogate and that of the original sequence match or differences between them are below of a given threshold. It was demonstrated in [31] that the procedure converges to produce a surrogate with the same power spectrum and distribution as the original series and a very good approximation of the original power spectrum with perfect preservation of the original distribution could be achieved after 100 iterations. The NCI computed over the original series was compared with the distribution of the NCI values calculated from the surrogate series. If the NCI computed over the original series was below the 5^th^ percentile of the NCI distribution calculated over the surrogates, the null hypothesis of linear dynamic was rejected and the alternative hypothesis of nonlinear dynamic was accepted. The rationale is that the deterministic nonlinear features, destroyed in the surrogates by the phase randomization procedure, were predicted better over the original series by the local nonlinear prediction method than over the surrogates. The percentage of nonlinear dynamics (NL%) was monitored over all the series and the indexes were labelled NL%_RR_, NL%_SAP_, NL%_DAP_, NL%_RESP_, NL%_MAP_, and NL%_MCBF_.

### Statistical analysis

Two-way analysis of variance (Holm-Sidak test for multiple comparisons) was applied to variability indexes to detect the effect of SAVR within the same experimental condition (i.e. REST or STAND) and the response to postural challenge within the same period of analysis (i.e. PRE or POST). A *p*<0.05 was always considered statistically significant. χ2 test (Fisher exact test) was applied over the categorical variables (i.e. presence/absence of nonlinear dynamics) to assess the effect of SAVR regardless of the posture and the influence of the postural challenge regardless of the period of analysis. If appropriate, the level of significance (i.e. 0.05) of the χ2 test was reduced according to the number of comparisons (i.e. 4) to account for the multiple comparison issue. Statistical analysis was carried out using a commercial statistical program (Sigmaplot, v.14.0, Systat Software, Inc., Chicago, IL, USA).

## Results

Table 2 reports the results of time and frequency domain analyses carried out over RR, SAP, DAP and RESP series at REST and during STAND in PRE and POST sessions. Regardless of the experimental condition (i.e. REST or STAND), μ_RR_ significantly decreased in POST compared to PRE. σ^2^_RR_ decreased and μ_DAP_ increased during POST with respect to PRE but the rise was significant solely at REST and during STAND respectively. No additional time domain markers were able to detect POST-PRE changes. The sole time domain index that was able to highlight a significant effect of the orthostatic challenge was μ_DAP_ that increased during STAND compared to REST in the POST session. None of the frequency domain markers, including f_RESP_ was able to separate either experimental conditions (i.e. REST and STAND) or periods of analysis (i.e. PRE and POST).

**Table 2:** Time and frequency domain parameters in PRE and POST at REST and during STAND over RR, SAP, DAP and RESP variabilities.

parameter	PRE		POST	
	REST	STAND	REST	STAND
μ_RR_ [ms]	944.1±98.4	860.3±103.7	722.8±128.3*	690.3±142.5*
σ^2^_RR_ [ms^2^]	937.4±1028.0	579.8±362.0	57.4±51.9*	62.3±63.8
HFa_RR_ [ms^2^]	172.9±249.8	114.8±220.1	20.5±35.5	17.1±16.5
μ_SAP_ [mmHg]	143.3±21.0	141.5±24.9	139.9±16.6	159.5±24.6
σ^2^_SAP_ [mmHg^2^]	23.2±18.5	33.7±39.4	26.8±19.4	32.9±16.0
LFa_SAP_ [mmHg^2^]	2.04±2.67	2.19±3.20	1.46±2.38	4.10±4.01
μ_DAP_ [mmHg]	68.6±20.2	77.9±18.2	75.9±15.0	97.6±21.9*§
σ^2^_DAP_ [mmHg^2^]	11.41±9.79	16.75±15.64	5.62±2.88	11.89±7.20
LFa_DAP_ [mmHg^2^]	2.45±3.76	3.05±4.50	0.87±1.15	2.63±3.18
f_RESP_ [Hz]	0.28±0.03	0.27±0.04	0.29±0.05	0.28±0.04

LF = low frequency; HF = high frequency; RR = heat period; μ_RR_ = RR mean; σ^2^_RR_ = RR variance; HFa_RR_ = HF power of RR expressed in absolute units; SAP = systolic arterial pressure; μ_SAP_ = SAP mean; σ^2^_SAP_ = SAP variance; LFa_SAP_ = LF power of SAP expressed in absolute units; DAP = diastolic arterial pressure; μ_DAP_ = DAP mean; σ^2^_DAP_ = DAP variance; LFa_DAP_ = LF power of DAP expressed in absolute units; f_RESP_ = respiratory rate derived from the RESP series; PRE = 1 day before surgery; POST = within 7 days after surgery; REST = at rest in supine position; STAND = during active standing. Results are reported as mean±standard deviation. The symbol * indicates *p*<0.05 versus PRE within the same experimental condition (i.e. REST or STAND). The symbol § indicates *p*<0.05 versus REST within the same acquisition session (i.e. PRE or POST).

Table 3 reports the results of time and frequency domain analyses carried out over MAP and MCBF series at REST and during STAND in PRE and POST sessions. None of time domain indexes was able to detect either the effect of the postural challenge or the impact of the cardiac surgery. The sole frequency domain markers modified by the cardiac surgery were HFa_MAP_ and HF%_MAP_: HFa_MAP_ increased in POST compared to PRE solely during STAND, while HF%_MAP_ raised in POST compared to PRE both at REST and during STAND.

**Table 3:** Time and frequency domain parameters in PRE and POST at REST and during STAND over MAP and MCBF variabilities.

parameter	PRE		POST	
	REST	STAND	REST	STAND
μ_MAP_ [mmHg]	98.7±14.3	97.2±21.0	93.6±16.4	117.5±21.4
σ^2^_MAP_ [mmHg^2^]	14.65±9.61	22.32±22.58	12.00±6.09	18.40±7.25
VLFa_MAP_ [mmHg^2^]	4.88±7.54	0.60±1.62	1.45±3.07	2.84±5.68
VLF%_MAP_	27.91±38.14	5.86±14.83	11.16±21.19	17.20±34.40
LFa_MAP_ [mmHg^2^]	2.91±2.71	5.54±6.60	1.66±1.32	1.10±2.21
LF%_MAP_	19.82±12.81	21.09±15.01	14.93±10.71	7.65±15.30
HFa_MAP_ [mmHg^2^]	1.74±1.50	1.63±1.90	3.26±3.16	6.82±6.97*
HF%_MAP_	12.66±8.49	8.41±4.04	25.45±13.93*	32.04±18.85*
μ_MCBF_ [cm·s^-1^]	44.39±15.34	36.15±13.79	46.38±13.90	30.00±9.15
σ^2^_MCBF_ [cm^2^·s^-2^]	11.49±15.27	12.51±13.61	11.52±7.31	22.42±27.55
VLFa_MCBF_ [cm^2^·s^-2^]	4.18±8.57	1.09±2.02	2.57±4.96	1.12±2.23
VLF%_MCBF_	21.99±30.60	12.21±22.61	17.64±34.37	1.77±3.54
LFa_MCBF_ [cm^2^·s^-2^]	1.93±3.25	3.71±7.93	2.90±5.97	3.81±6.40
LF%_MCBF_	17.63±12.41	19.56±17.54	16.45±30.70	12.63±11.58
HFa_MCBF_ [cm^2^·s^-2^]	1.95±3.07	2.92±2.91	1.62±1.31	3.17±4.81
HF%_MCBF_	13.49±7.74	24.89±10.39	12.97±9.74	11.92±9.16

LF = low frequency; VLF = very LF; HF = high frequency; MAP = mean arterial pressure; μ_MAP_ = MAP mean; σ^2^_MAP_ = MAP variance; VLFa_MAP_ and VLF%_MAP_ = VLF power of MAP expressed in absolute and percent units; LFa_MAP_ and LF%_MAP_ = LF power of MAP expressed in absolute and percent units; HFa_MAP_ and HF%_MAP_ = HF power of MAP expressed in absolute and percent units; MCBF = mean cerebral blood flow; μ_MCBF_ = MCBF mean; σ^2^_MCBF_ = MCBF variance; VLFa_MCBF_ and VLF%_MCBF_ = VLF power of MCBF expressed in absolute and percent units; LFa_MCBF_ and LF%_MCBF_ = LF power of MCBF expressed in absolute and percent units; HFa_MCBF_ and HF%_MCBF_ = HF power of MCBF expressed in absolute and percent units; PRE = 1 day before surgery; POST = within 7 days after surgery; REST = at rest in supine position; STAND = during active standing. Results are reported as mean±standard deviation. The symbol * indicates *p*<0.05 versus PRE within the same experimental condition (i.e. REST or STAND).

**Fig 1:**
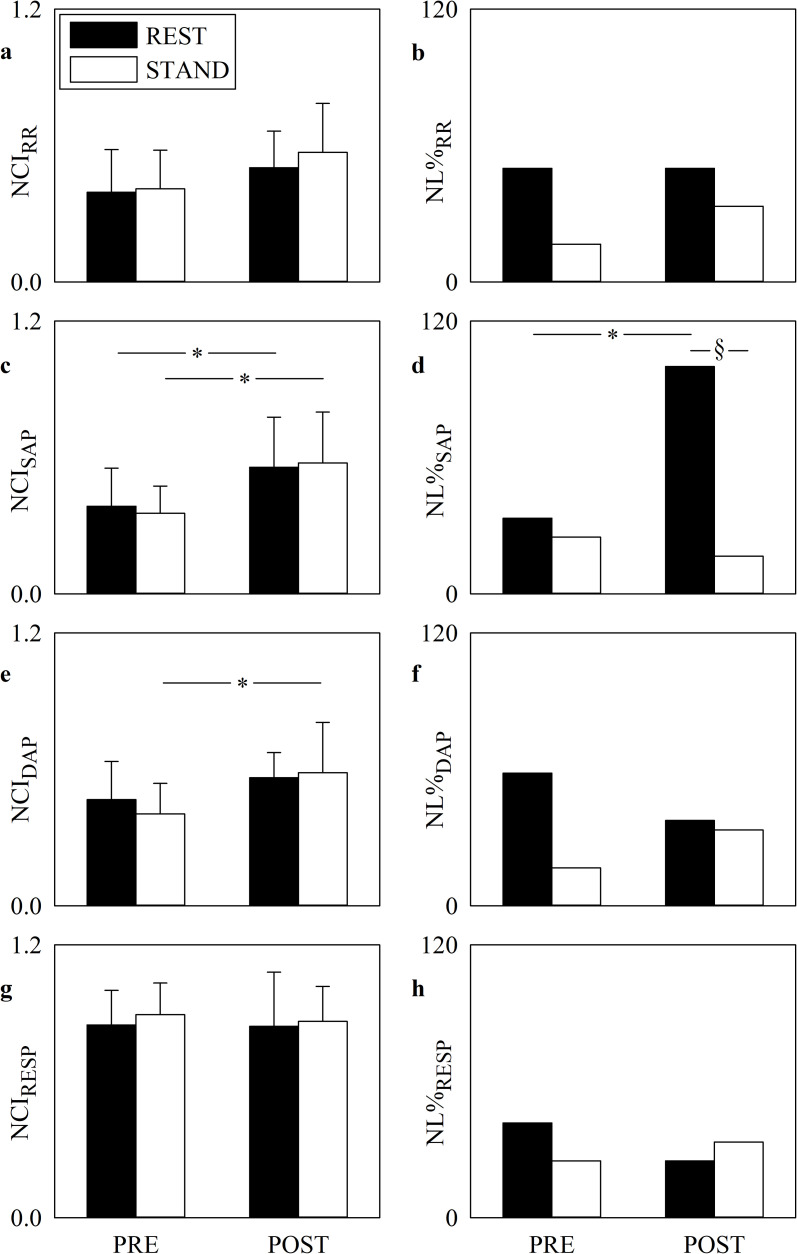
Complexity and nonlinearity of RR, SAP, DAP and RESP series. The grouped error bar graphs show NCI_RR_ (a), NCI_SAP_ (c), NCI_DAP_ (e) and NCI_RESP_ (g), while the grouped bar graphs show NL%_RR_ (b), NL%_SAP_ (d), NL%_DAP_ (f) and NL%_RESP_ (h) in PRE and POST. The solid black and white bars are relevant to markers computed at REST and during STAND respectively. Data in the grouped error bar graphs are given as mean plus standard deviation. The symbol * indicates a significant modification between PRE and POST within the same experimental condition with *p*<0.05, while the symbol § indicates the significant REST-STAND variation within the same period of analysis with *p*<0.05.

The grouped error bar graphs of Fig 1 show the NCI computed over RR (Fig 1A), SAP (Fig 1C), DAP (Fig 1E) and RESP (Fig 1G) series, while the grouped bar graphs of Fig 1 show the NL% over RR (Fig 1B), SAP (Fig 1D), DAP (Fig 1F) and RESP (Fig 1H) series during PRE and POST sessions. Markers were computed at REST (solid black bars) and during STAND (solid white bars). NCI_RR_ and NCI_RESP_ did not vary in response to cardiac surgery. NCI_SAP_ rose significantly after SAVR both at REST and during STAND and the POST-PRE increase of NCI_DAP_ was significant only during STAND. Within the same period of analysis (i.e. PRE or POST) posture modification did not affect any NCI marker. NL%_SAP_ was significantly higher at REST in the POST session compared to PRE and lower in POST session during STAND compared to REST. NL%_RR_, NL%_DAP_ and NL%_RESP_ did not vary with postural challenge and SAVR surgery.

**Fig 2:**
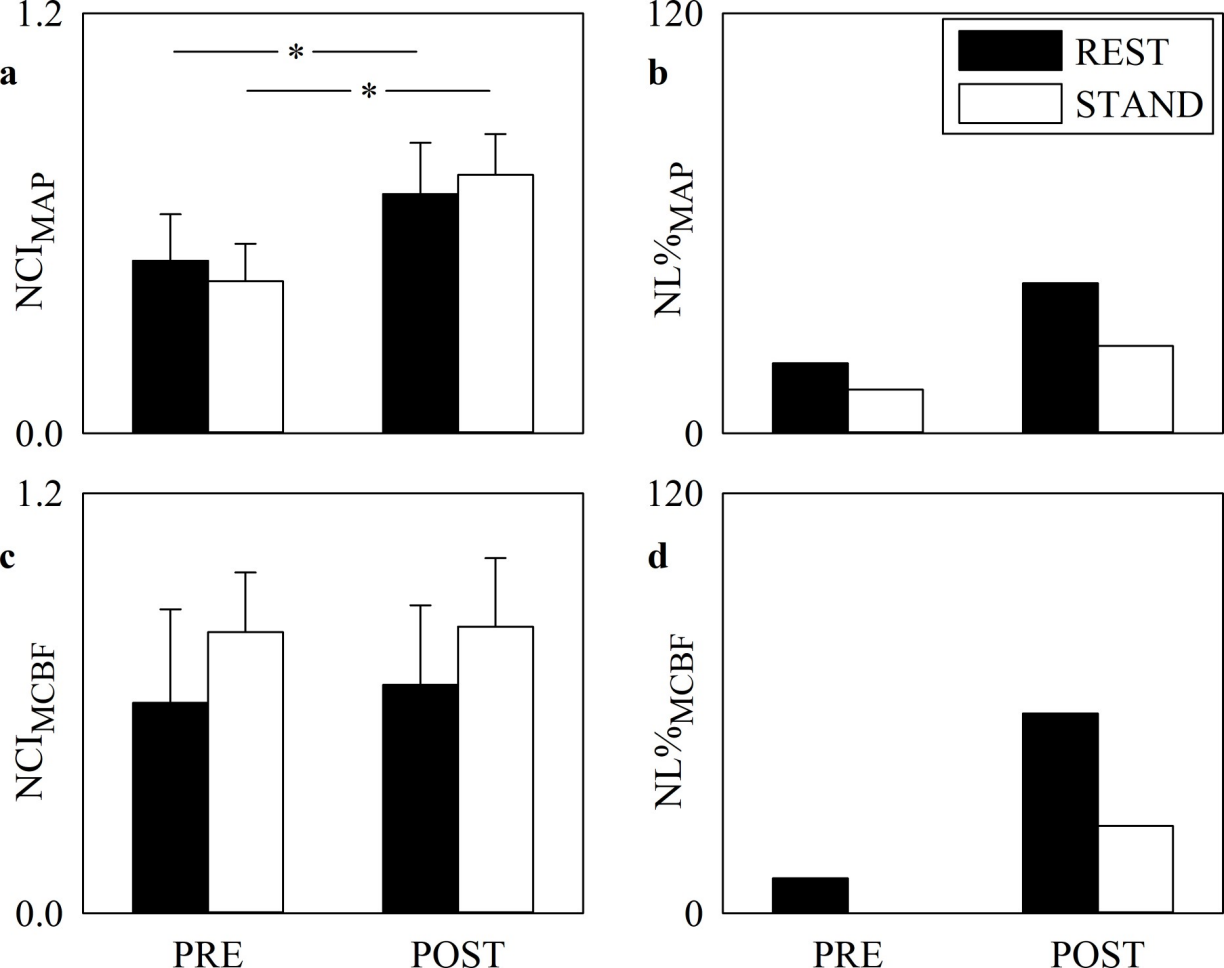
Complexity and nonlinearity of MAP and MCBF series. The grouped error bar graphs show NCI_MAP_ (a) and NCI_MCBF_ (c), while the grouped bar graphs show NL%_MAP_ (b) and NL%_MCBF_ (d) in PRE and POST. The solid black and white bars are relevant to markers computed at REST and during STAND respectively. Data in the grouped error bar graphs are given as mean plus standard deviation. The symbol * indicates a significant modification between PRE and POST within the same experimental condition with *p*<0.05.

Fig 2 has the same structure of Fig 1 but it shows the NCI computed over MAP (Fig 2A) and MCBF (Fig 2C) series and NL% over MAP (Fig 2B) and MCBF (Fig 2D) series during PRE and POST sessions. NCI_MCBF_ did not vary in response to cardiac surgery. NCI_MAP_ rose significantly after SAVR both at REST and during STAND. Within the same period of analysis (i.e. PRE or POST) posture did not affect any NCI marker. NL%_MAP_ and NL%_MCBF_ did not vary with postural challenge and SAVR surgery.

When all series were pooled together regardless of the period of analysis, NL% was significantly higher at REST than during STAND. Conversely, when all series were pooled together regardless of the experimental condition, no effect of the surgery over NL% was detectable.

**Fig 3:**
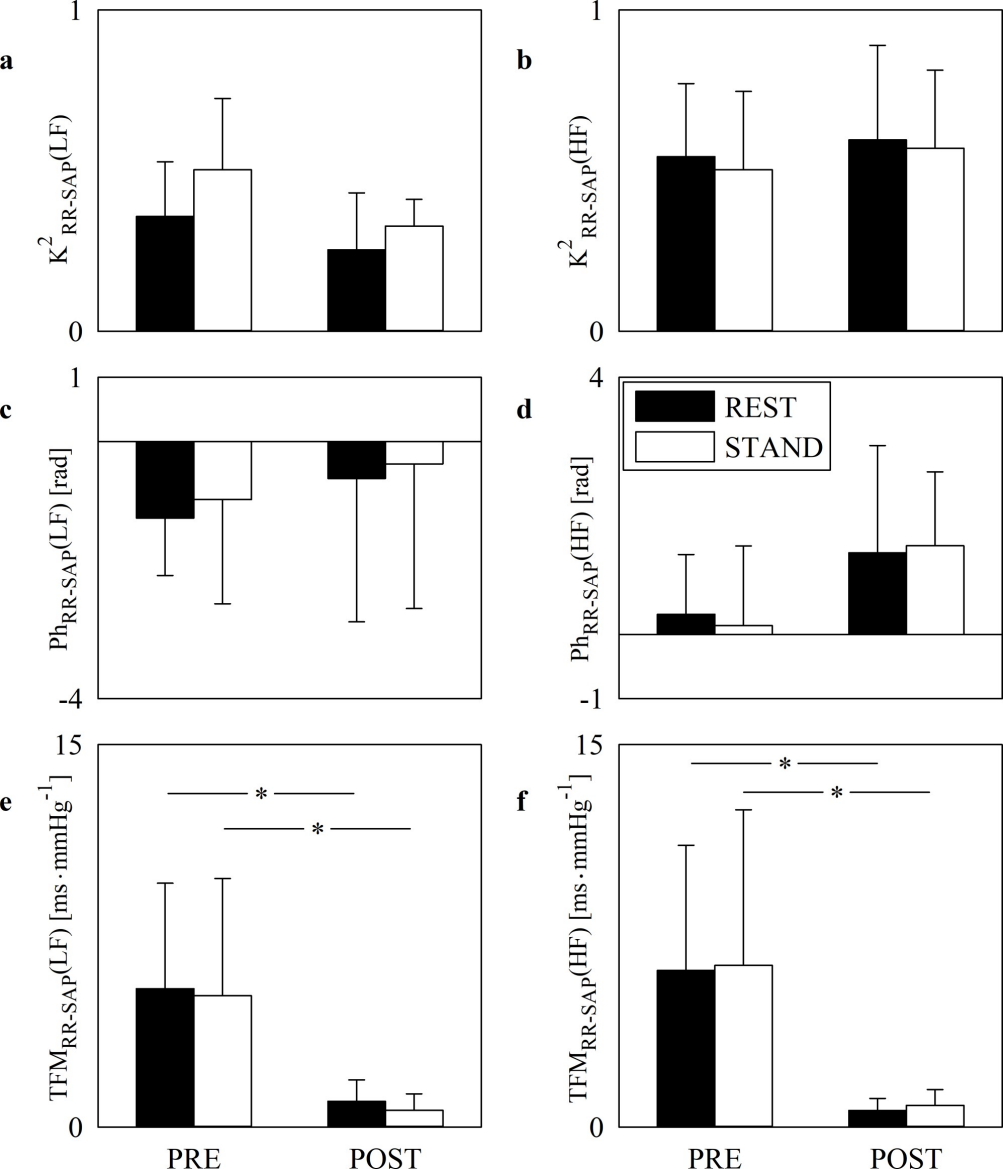
Cross-spectral analysis of RR and SAP series. The error bar graphs show K^2^_RR-SAP_(LF) (a), K^2^_RR-SAP_(HF) (b), Ph_RR-SAP_(LF) (c), Ph_RR-SAP_(HF) (d), TFM_RR-SAP_(LF) (e) and TFM_RR-SAP_(HF) (f) in PRE and POST. The solid black and white bars are relevant to bivariate frequency domain markers computed at REST and during STAND respectively. The horizontal solid line in (c) and (d) marks the null phase. Data are given as mean plus standard deviation. The symbol * indicates a significant modification between PRE and POST within the same experimental condition with *p*<0.05.

The grouped error bar graphs of Fig 3 show K^2^_RR-SAP_(LF) (Fig 3A), K^2^_RR-SAP_(HF) (Fig 3B), Ph_RR-SAP_(LF) (Fig 3C), Ph_RR-SAP_(HF) (Fig 3D), TFM_RR-SAP_(LF) (Fig 3E) and TFM_RR-SAP_(HF) (Fig 3F) during PRE and POST sessions. Markers were computed at REST (solid black bars) and during STAND (solid white bars). Orthostatic stimulus and cardiac surgery did not influence either K^2^ or phase markers. Regardless of the frequency band (i.e. LF or HF) SAVR surgery decreased TFM both at REST and during STAND. Assigned the period of analysis (i.e. PRE or POST) the impact of postural maneuver on the baroreflex sensitivity as measured via TFM was negligible in both LF and HF bands.

**Fig 4:**
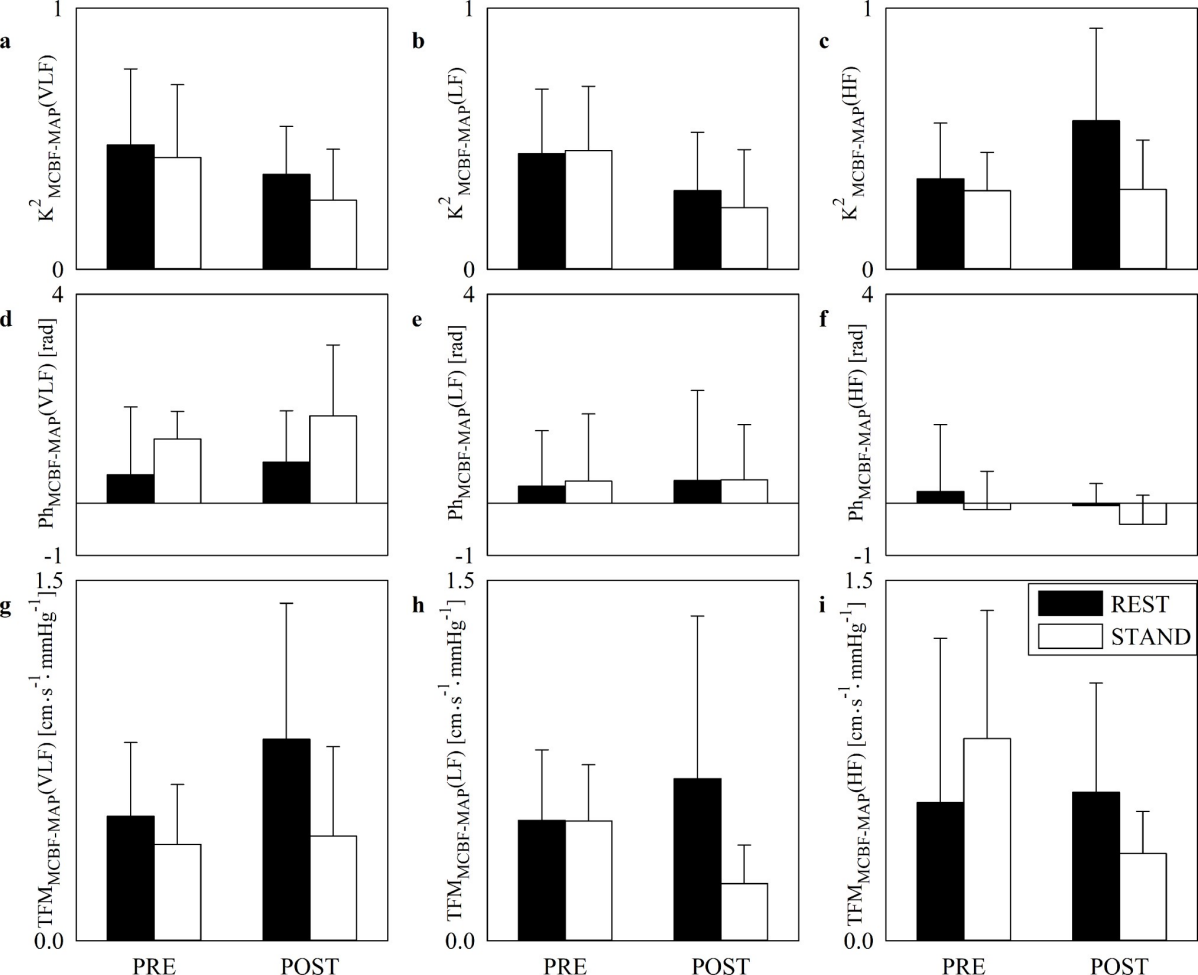
Cross-spectral analysis of MCBF and MAP series. The error bar graphs show K^2^_MCBF-MAP_(VLF) (a), K^2^_MCBF-MAP_(LF) (b), K^2^_MCBF-MAP_(HF) (c), Ph_MCBF-MAP_(VLF) (d), Ph_MCBF-MAP_(LF) (e), Ph_MCBF-MAP_(HF) (f), TFM_MCBF-MAP_(VLF), (g) TFM_MCBF-MAP_(LF) (h) and TFM_MCBF-MAP_(HF) (i) in PRE and POST. The solid black and white bars are relevant to bivariate frequency domain markers computed at REST and during STAND respectively. The horizontal solid line in (d), (e) and (f) marks the null phase. Data are given as mean plus standard deviation.

Fig 4 has the same structure as Fig 3 but it shows K^2^_MCBF-MAP_(VLF) (Fig 4A), K^2^_MCBF-MAP_(LF) (Fig 4B), K^2^_MCBF-MAP_(HF) (Fig 4C), Ph_MCBF-MAP_(VLF) (Fig 4D), Ph_MCBF-MAP_(LF) (Fig 4E), Ph_MCBF-MAP_(HF) (Fig 4F), TFM_MCBF-MAP_(VLF) (Fig 4G), TFM_MCBF-MAP_(LF) (Fig 4H) and TFM_MCBF-MAP_(HF) (Fig 4I) during PRE and POST sessions. Regardless of the frequency band (i.e. VLF, LF or HF) K^2^, phase and TFM of the MCBF-MAP dynamical relation did not vary with posture and SAVR surgery.

## Discussion

The major findings can be summarized as follows: i) orthostatic maneuver did not modify cardiovascular regulatory indexes in PRE, thus suggesting that SAVR patients featured a depressed vagal autonomic and baroreflex controls just before the cardiac surgery; ii) vagal autonomic and cardiac baroreflex impairment were observed after SAVR; iii) cerebrovascular variability and cerebral autoregulation appeared to be less affected by SAVR; iv) at the vascular control level the autonomic control impairment took the form of a post-surgery increased complexity of SAP, DAP and MAP variabilities and an augmented presence of nonlinear dynamics of SAP after SAVR; v) STAND reduced the likelihood of finding nonlinear dynamics; vi) respiratory rate was not affected by either SAVR surgery or orthostatic challenge.

### Effect of STAND on a cohort of patients undergoing SAVR surgery

Orthostatic challenges did not provoke the expected decrease of vagal modulation directed to the heart [1, 3, 4, 6, 49], the expected increase of sympathetic modulation directed to the vessels [4, 6] and the expected diminution of baroreflex sensitivity [4, 43, 50, 51]. Indeed, STAND induced negligible effects over cardiovascular markers in PRE. This result can be taken as a hallmark of cardiovascular control dysfunction in our cohort of patients just before SAVR surgery. The effect of STAND on cerebrovascular variability and cerebral autoregulation is analogously limited. This result might suggest a possible impairment of the cerebrovascular control mechanisms given that a significant increase of the MCBF-MAP coupling strength was found when this marker of the dynamical MCBF-MAP relation was assessed in a healthy group [35].

### Effect of SAVR on the amplitude of cardiovascular and cerebrovascular variability markers

It is well-known that SAVR reduced cardiac vagal control [22–26]. This observation is confirmed in this study by the dramatic postoperative reduction of σ^2^_RR_ observed at REST. This observation is important because our subjects exhibited an impaired cardiac vagal control already in PRE as indicated by the negligible STAND-REST variation of the HFa_RR_ power [1, 3, 4, 6, 49]. The post-surgery depression of vagal regulation might expose our group to possible postoperative arrhythmic events [26, 27]. The unaltered value of σ^2^_SAP_, σ^2^_DAP_ and σ^2^_MAP_ during POST regardless of the experimental condition points toward a more preserved sympathetic control directed to the vessels. These findings are compatible with the postoperative pharmacological therapy as well as the physical debilitation and the high perceived level of stress associated to the POST condition. The amplitude of the MCBF fluctuations seems to be preserved as indicated by the invariable amount of σ^2^_MCBF_ and the unchanged MCBF powers in the various frequency bands in POST compared to PRE. This finding could be related to a better preservation of the sympathetic control after SAVR. Indeed, MCBF regulation is under sympathetic control [21], even though different mechanisms might contribute to preserve cerebral autoregulation including the postoperative improvement of the valve hemodynamics and aortic blood flow profile [17].

### Effect of SAVR on the complexity and nonlinearities of cardiovascular and cerebrovascular variabilities

In spite of the maintenance of the magnitude of the SAP, DAP and MAP oscillations, important modifications of the SAP, DAP and MAP dynamics were observed. Indeed, complexity of SAP, DAP and MAP variability increased after SAVR and this increase was accompanied by a significant rise of SAP nonlinear dynamics, especially visible at REST. Even a tendency toward an increased likelihood of observing nonlinearities in MCBF series was visible in POST. We conclude that even sympathetic control seems to be affected by SAVR. An increased SAP variability complexity could be attributed to the inability of the sympathetic control to produce synchronous oscillations of peripheral resistances by producing rhythmical alternations between suitable vasoconstriction and vasodilatation episodes [52] as suggested in some pathological situations [7]. It is worth noting that, in spite of the limited response to STAND, we observed the typical decrease of the likelihood of detecting nonlinear dynamics in cardiovascular variability reported in healthy individuals during orthostatic challenge [9, 10], thus indicating a residual cardiovascular control in both PRE and POST.

### Effect of SAVR on the baroreflex control and cerebral autoregulation markers

Markers of cardiac baroreflex and cerebral autoregulation were evaluated via bivariate frequency domain metrics assessing phase and TFM from an input to an output and their degree of association via K^2^ [32]. In the case of the assessment of the cardiac baroreflex the input is SAP variability and the output is the RR variability [40, 42–44], while in the case of the evaluation of cerebral autoregulation the input is MAP variability and the output is the MCBF variability [18, 45, 46]. It is well-known that SAVR reduced baroreflex control [26]. This observation is confirmed in this study by the dramatic postoperative reduction of TFM_RR-SAP_ in the LF and HF bands both at REST and during STAND. Signs of an impaired cardiac baroreflex were observable just before SAVR given that the expected decrease of TFM_RR-SAP_ in the LF and HF bands during STAND compared to REST in PRE was not visible [4, 43, 50, 51]. The post-surgery depression of the baroreflex regulation in association with the weakness of this reflex just in PRE might expose SAVR patients to possible postoperative arrhythmic events [26, 27]. Cerebral autoregulation mechanisms seem to be preserved after SAVR as indicated by the negligible post-surgery changes of TFM, phase and K^2^ between MCBF and MAP in the various frequency bands and this result is tightly linked to the preservation of the MAP and MCBF powers. Again, this finding could be related to the maintenance of the sympathetic control after SAVR, even though the contribution of different factors including the beneficial effect of surgery over the arterial blood flow profile cannot be dismissed. This finding suggests that SAVR patient might be more at risk of developing postoperative arrhythmic episodes than postoperative cerebrovascular adverse events.

## Conclusions

The major depression of the vagal autonomic control and cardiac baroreflex after SAVR might expose SAVR subjects to a greater risk of postoperative arrhythmic adverse events. Cerebrovascular regulation seems to be less affected by post-surgery autonomic regulation derangement and this finding suggests that SAVR patients feature internal resources that might maintain the risk of postoperative cerebrovascular adverse events at the preoperative level. We remark that the direct association between cardiovascular adverse events after SAVR and the depression of vagal control and cardiac baroreflex regulation deserves specific future studies involving an adequate number of subjects and classification of type and rate of events. Given the important dispersion of the cerebrovascular variability and cerebral autoregulation indexes, individual analysis is necessary to check whether people with markers compatible with a cerebrovascular impairment might have a greater probability of developing postoperative cerebrovascular adverse events. We remark the need of increasing the power of the study by enlarging the size of the group to verify whether the inability to distinguish differences could the mere consequence of the small sample size. Future studies should assess whether indexes could indicate any crosstalk between cardiovascular and cerebrovascular regulations and could be helpful to identify risky subjects.

## Supporting information

S1 Appendix Spectral and cross-spectral methods. It contains supporting information relevant to spectral and cross-spectral methods.
(PDF)

S1 Dataset Database of the extracted variability markers. It contains spectral, cross-spectral, complexity indexes as well as the result of the test checking for the presence of nonlinear dynamics computed at REST and during STAND before and after SAVR.
(ZIP)
