# Supplementary material for: Evaluation of the impact of surgical aortic valve replacement on short-term cardiovascular and cerebrovascular controls through spontaneous variability analysis
Source: PLoS One. 2020 Dec 10;15(12):e0243869. doi: 10.1371/journal.pone.0243869 (PMC7728248; doi:10.1371/journal.pone.0243869)
Supplement: S1 Appendix — It contains supporting information relevant to spectral and cross-spectral methods. (PDF) [file pone.0243869.s001.pdf]

Appendix of the MS "Evaluation of the impact of surgical aortic valve replacement on short-term cardiovascular and cerebrovascular controls through spontaneous variability analysis" by Alberto Porta, Angela Fantinato, Vlasta Bari, Francesca Gelpi, Beatrice Cairo, Beatrice De Maria, Enrico Giuseppe Bertoldo, Valentina Fiolo, Edward Callus, Carlo De Vincentiis, Marianna Volpe, Raffaella Molfetta, and Marco Ranucci, PLoS ONE

A.1. Univariate model-based frequency domain analysis

Univariate model-based frequency domain analysis was performed via a parametric approach exploiting the autoregressive (AR) model [1,2]. Briefly, the AR model describes the zero-mean series $y=\{y_n, n=1, \ldots, N\}$, where n is the progressive sample counter and N is the series length, as

$$y_n = \sum_{k=1}^{p} a_k \cdot y_{n-k} + w_n \,, \qquad (1)$$

where a_k with $k=1, \ldots, p$ are real coefficients and w is a realization of zero-mean Gaussian white noise with variance λ^2. The series y is the sum of two parts: i) a fully predictable portion modeled as a linear combination of p past samples weighted by real coefficients; ii) a fully unpredictable portion described by w. The equivalent representation of the AR process in the z-domain allows one to write the z-transformation of the AR process as the product of the z-transformation of w by the function

$$H(z) = \frac{1}{1-\sum_{k=1}^{p} a_k \cdot z^{-k}} = \frac{z^p}{\prod_{k=1}^{p}(z-p_k)} \,, \qquad (2)$$

referred to as transfer function of the AR process, featuring p singularities $p_k=\rho e^{j\varphi}$ with $k=1, \ldots, p$ called poles where j is the imaginary unit in the complex plane. Given that the coefficients of the AR process are real, if a complex pole (i.e. $p_k=\rho e^{j\varphi}$ with $\varphi\neq 0$ and $\varphi\neq\pi$) is present, the conjugate $p_k=\rho e^{-j\varphi}$ is found as well. The Levinson-Durbin recursive algorithm [2,3] was utilized to estimate directly from y, the coefficients a_k with $k=1, \ldots, p$ and λ^2. The model order p is decided with some figure of merit, for example the Akaike information criterion [4]. According to the maximum entropy spectral estimation approach [2,3], the power spectral density $S(f)$ can be computed from $H(z)$ and λ^2 as

$$S(f) = T \cdot H(z) \cdot H(z^{-1}) \cdot \lambda^2 \Big|_{z=e^{j2\pi fT}} \,, \qquad (3)$$

where T is the sampling period. $S(f)$ can be factorized in a sum of terms [5], referred to as spectral components that, transformed back in the time domain, correspond to basic AR processes associated to a real pole or a pair of complex and conjugate poles of $H(z)$. The sum of the power of all components is equal to the variance of the AR process. A spectral component was attributed to a given frequency band if the phase φ of the associated real pole or pair of complex and conjugate poles, when converted into a frequency f using the transformation $f=\varphi\cdot(2\pi T)^{-1}$, dropped in that frequency band. The total power in a given frequency band was computed as the sum of the powers of all spectral components attributed to that band and the power of each spectral component was efficiently computed via the residue theorem [5].

A.2. Bivariate model-based frequency domain analysis

Bivariate model-based frequency domain analysis of $y_1=\{y_{1,n}, n=1, \ldots, N\}$ and $y_2=\{y_{2,n}, n=1, \ldots, N\}$ was performed via a parametric approach exploiting the bivariate AR model [1,5,6]. Briefly, the bivariate AR model jointly describes y_1 and y_2 as

$$y_{1,n} = \sum_{k=1}^{p} a_{11,k} \cdot y_{1,n-k} + \sum_{k=\tau_{12}}^{p} a_{12,k} \cdot y_{2,n-k} + w_{1,n} \quad (4)$$

$$y_{2,n} = \sum_{k=\tau_{21}}^{p} a_{21,k} \cdot y_{1,n-k} + \sum_{k=1}^{p} a_{22,k} \cdot y_{2,n-k} + w_{2,n} \,, \quad (5)$$

where $a_{11,k}$ and $a_{22,k}$ with k=1, …, p are the real coefficients of the auto-regressions of y_1 and y_2 on its own past respectively, $a_{12,k}$ with $k=\tau_{12}$, …, p and $a_{21,k}$ with $k=\tau_{21}$, …, p are the real coefficients of the cross-regressions of y_1 on past values of y_2 and *vice versa*, and w_1 and w_2 are two uncorrelated zero-mean white noises with variance $\lambda^2{}_1$ and $\lambda^2{}_2$ respectively. τ_{12} and τ_{21} represent the delay of interactions from y_2 to y_1 and from y_1 to y_2 respectively. The equivalent representation of the bivariate AR process $\boldsymbol{y}=|y_1\ y_2|'$ in the z-domain allows one to provide the z-transformation of $\boldsymbol{y}$ as the product the z-transformation of the white noise $\boldsymbol{w}=|w_1\ w_2|'$ by the transfer function matrix $\boldsymbol{H}(z)=(\mathbf{I}-\mathbf{A}(z))^{-1}$, where $\mathbf{I}$ is the identity 2x2 matrix and

$$\boldsymbol{A}(z) = \begin{vmatrix} \sum_{k=1}^{p} a_{11,k} \cdot z^{-k} & \sum_{k=\tau_{12}}^{p} a_{12,k} \cdot z^{-k} \\ \sum_{k=\tau_{21}}^{p} a_{21,k} \cdot z^{-k} & \sum_{k=1}^{p} a_{22,k} \cdot z^{-k} \end{vmatrix} \quad (6)$$

is the 2x2 matrix of polynomials in z and the symbol $'$ denotes the transpose operator. The coefficients of $\boldsymbol{A}(z)$ are estimated directly from $\boldsymbol{y}$ via the traditional least squares approach solved using Cholesky decomposition method [2,3]. The number of useful past samples p is usually fixed *a priori* according to methodological considerations about spectral resolution or optimized using some figure of merit, for example the Akaike information criterion extended to bivariate processes [4] and the delays τ_{12} and τ_{21} were usually set to 1 when cross-spectral features were estimated [5]. According to the maximum entropy spectral estimation approach [2,3], power spectral density matrix $\boldsymbol{S}(f)$ can be computed from $\boldsymbol{H}(z)$ and the covariance matrix $\boldsymbol{\Lambda}$ of $\boldsymbol{w}$ as

$$\boldsymbol{S}(f) = T \cdot \boldsymbol{H}(z) \cdot \boldsymbol{\Lambda} \cdot \boldsymbol{H}'(z^{-1}) \Big|_{z=e^{j2\pi fT}} \,. \quad (7)$$

$\boldsymbol{S}(f)$ features the power spectral densities of y_1 and y_2, $S_{11}(f)$ and $S_{22}(f)$, on the main diagonal and the cross-spectral densities from y_2 to y_1 and from y_1 to y_2, $S_{12}(f)$ and $S_{21}(f)$, out of the main diagonal. While $S_{11}(f)$ and $S_{22}(f)$ are real functions of f, cross-spectral densities are complex functions of f with $S_{12}(f)=S^*_{21}(f)$, where the symbol * denotes the complex conjugation operator. The transfer function from y_2 to y_1 is $H_{12}(f)=S_{12}(f)/S_{22}(f)$ [7]. The transfer function modulus from y_2 to y_1 is $|H_{12}(f)|=|S_{12}(f)|/S_{22}(f)$, where $|\cdot|$ takes the modulus of $S_{12}(f)$. The phase of $H_{12}(f)$ is coincident with the phase of $S_{12}(f)$. It ranges from $-\pi$ to $+\pi$ and it is known at multiples of 2π. This means that, if the phase of $H_{12}(f)$ is $\angle H_{12}(f)$, also $\angle H_{12}(f)+2\pi k$ with $k=0, \pm1, \pm2, \ldots$ is admissible leading to the ambiguous conversion of phase values into delays or advancements [8,9] that might be solved using *a priori* information about the latency of interactions [8,9]. Squared coherence function $K^2{}_{12}(f)=|S_{12}(f)|^2/[S_{11}(f)\cdot S_{22}(f)]$ [7] is a measure of the strength of linear association between y_1 and y_2 as a function of f. $K^2{}_{12}(f)$ ranges from 0 to 1, where 0 indicates null and maximum linear association between y_1 and y_2 respectively. $K^2{}_{12}(f)$ is a symmetric function [i.e. $K^2{}_{12}(f)=K^2{}_{21}(f)$] [7].

References

[1] Porta A, Baselli G, Cerutti S. Implicit and explicit model-based signal processing for the analysis of short term cardiovascular interactions, Proc IEEE. 2006; 94: 805-818.

[2] Kay SM, Marple SL, Spectrum analysis: a modern perspective, Proc. IEEE. 1981; 69: 1380-1418.

[3] Söderström T, Stoica P. System Identification, Englewood Cliffs, UK: Prentice Hall International; 1989.

[4] Akaike H. A new look at the statistical novel identification, IEEE Trans Autom Control. 1974; 19: 716-723.

[5] Baselli G, Porta A, Rimoldi O, Pagani M, Cerutti S. Spectral decomposition in multichannel recordings based on multi-variate parametric identification, IEEE Trans Biomed Eng. 1997; 44: 1092-1101.

[6] Porta A, Baselli G, Rimoldi O, Malliani A, Pagani M. Assessing baroreflex gain from spontaneous variability in conscious dogs: role of causality and respiration, Am J Physiol. 2000; 279: H2558-H2567.

[7] Porta A, Furlan R, Rimoldi O, Pagani M, Malliani A, van de Borne P. Quantifying the strength of linear causal coupling in closed loop interacting cardiovascular variability series, Biol Cybern. 2002; 86: 241-251.

[8] Porta A, Catai AM, Takahashi ACM, Magagnin V, Bassani T, Tobaldini E, et al. Causal relationships between heart period and systolic arterial pressure during graded head-up tilt, Am J Physiol. 2011; 300: R378-R386.

[9] Milan-Mattos JC, Porta A, Perseguini NM, Minatel V, Rehder-Santos P, Takahashi ACM, et al. Influence of age and gender on the phase and strength of the relation between heart period and systolic blood pressure spontaneous fluctuations, J Appl Physiol. 2018; 124: 791–804.
