# Supplementary material for: Evaluation of the impact of surgical aortic valve replacement on short-term cardiovascular and cerebrovascular controls through spontaneous variability analysis
Source: PLoS One. 2020 Dec 10;15(12):e0243869. doi: 10.1371/journal.pone.0243869 (PMC7728248; doi:10.1371/journal.pone.0243869)
Supplement: S1 Dataset — It contains spectral, cross-spectral, complexity indexes as well as the result of the test checking for the presence of nonlinear dynamics computed at REST and during STAND before and after SAVR. (ZIP) [file pone.0243869.s002.zip › legenda.doc]

BRS: file containing baroreflex markers

CA: file containing cerebral autoregulation markers

HP_NL: file containing nonlinear and complexity analysis markers over heart period series

MAP_NL: file containing nonlinear and complexity analysis markers over mean arterial pressure series

SAP_NL: file containing nonlinear and complexity analysis markers over systolic arterial pressure series

DAP_NL: file containing nonlinear and complexity analysis markers over diastolic arterial pressure series

MCBF_NL: file containing nonlinear and complexity analysis markers over mean cerebral blood flow series

R_NL: file containing nonlinear and complexity analysis markers over respiratory series

HP_PSD: file containing spectral markers over heart period series

MAP_PSD: file containing spectral markers over mean arterial pressure series

SAP_PSD: file containing spectral markers over systolic arterial pressure series

DAP_PSD: file containing spectral markers over diastolic arterial pressure series

MCBF_PSD: file containing spectral markers over mean cerebral blood flow series

R_PSD: file containing spectral markers over respiratory series
